# An Outbreak of *Serratia marcescens* in a Moroccan Neonatal Intensive Care Unit

**DOI:** 10.1155/2018/4867134

**Published:** 2018-11-01

**Authors:** Abdellatif Daoudi, Fatiha Benaoui, Nadia El Idrissi Slitine, Nabila Soraa, Fadl Mrabih Rabou Maoulainine

**Affiliations:** ^1^Neonatal Intensive Care Unit, Mother-Child Center, Mohammed VI University Medical Hospital of Marrakesh, Morocco; ^2^Research Team Childhood, Health and Development, Faculty of Medicine and Pharmacy Cadi Ayyad University, Morocco; ^3^Biology Laboratory, Mother-Child Unit, Mohammed VI University Medical Hospital of Marrakesh, Morocco

## Abstract

*Serratia marcescens* (*S. marcescens*) is an *Enterobacteriaceae* microorganism that is widespread in the environment, which may be the source of nosocomial infections, rare in the newborn but severe, and often in the form of outbreaks. The aim of our study is to report our experience, during an outbreak of *S. marcescens*, to show the severity of this germ, with review of the literature. Our study was retrospective, including 8 newborns with *S. marcescens* nosocomial infection, collected in the neonatal intensive care unit of Mohammed VI University Medical Hospital, during the epidemic period, over a period of 2 months (July and August 2016). The mean gestational age of the cases was 36 weeks of amenorrhea. Boys accounted for 75% of the cases. The average weight was 1853 grams. All the patients were initially placed under empiric antibiotic therapy based on ceftriaxone and gentamicin. The mean duration of nosocomial infection, diagnosed in all cases by blood cultures, was 7 days. The strains of *S. marcescens* were in 75% of the cases sensitive to the cephalosporins, intermediate sensitivity in 12.5% of cases and resistant in 12.5% of cases. The outcome was fatal in 62.5% of cases. *S. marcescens* nosocomial infections are often reported on epidemic series, and their eradication is not always easy.

## 1. Introduction

Nosocomial infections in neonatal intensive care unit still represent a public health problem by the morbidity and mortality that they engender. The most incriminated pathogens are *Enterobacteriaceae* that belong to the Gram-negative bacillus (GNB) family, especially in developing countries, and they can represent 51.5% of the cases [1]. Among the GNB, *Serratia marcescens (S. marcescens)* is a ubiquitous opportunistic pathogen, whose eradication of the environment is very difficult [2]. Nosocomial infections with *S. marcescens* are rare in newborns and often described as outbreaks [2, 3]. Infection can occur in the newborn as pneumonia, bacteremia, conjunctivitis, urinary tract infections, and even gastroenteritis. The outcome of these infections is often serious, with a heavy morbidity and mortality; whence, the importance of initiating, as soon as the diagnosis of this germ is made, controls measures before the propagation which can be spectacular.

The aim of this study is to share our experience of nosocomial infections due to this germ, to show the gravity of this germ, with a review of the literature.

## 2. Patients and Methods

This is a descriptive retrospective study, performed in the neonatal intensive care unit (NICU) of Mohammed VI University Medical Hospital, Marrakesh, Morocco. During the epidemic period of this germ, July and August 2016, the population in our study included neonates hospitalized in the NICU, whose postnatal age ranged from 0 to 28 days with the diagnosis of nosocomial infection of S. marcescens, which was made by the positivity of bacteriological samples at least 48 hours after admission to our NICU. Data collection was done using an individual record.

The literature review was done on PubMed including writings describing *Serratia marcescens* outbreaks in neonates in the NICU.

## 3. Results

We collected eight cases, which were included during the study period, July and August 2016, which corresponded to the epidemic period of *S. marcescens* bacteremia.

The epidemic began in early July 2016 in a premature 33.9 GA admitted for hyaline membrane disease, which showed after 3 days of his hospitalization signs of sepsis with isolation of *Serratia marcescens* in the blood culture, and other cases were diagnosed throughout the July period.

The mean gestational age of our patients was 36 weeks (wk), with extremes ranging from 33.2 to 40.8 wk. Newborns were premature in 75% of cases. The sex ratio (boy/girl) was 3. The weight ranged from 1130 grams to 3600 grams with an average weight of 1853 grams. Hyaline membrane disease was the most diagnosis of hospitalization in 62.5% of cases and neonatal pulmonary infection in 37.5% of cases (Table 1).

On admission, all patients were treated with ceftriaxone and gentamicin antibiotics, and 87.5% of cases were artificially ventilated. The diagnosis of nosocomial infection was made over an average of 7 days of hospitalization, with extremes ranging from 3 days to 12 days. The diagnosis was made in the presence of clinical signs and/or biological abnormalities on the hemogram or ascension C-reactive protein with *S. marcescens-*positive blood cultures. Isolated *S. marcescens* strains were susceptible in 75% of cases to 3rd generation cephalosporins and all susceptible to imipenem, to ciprofloxacin, and to aminoglycosides (amikacin and gentamicin), but all were resistant to colistin (Table 2). After diagnosis of nosocomial *S. marcescens* infection, all patients were treated with imipenem and amikacin. The outcome was favorable in 37.8% of the cases, and the death was reported in 62.5%. The average hospital stay was 22.75 days, with extremes ranging from 12 days to 34 days.

## 4. Discussion

Nosocomial infections in neonatal intensive care units are a public health problem because of their heavy consequences on morbidity and mortality and the important cost they generate [1]. *S. marcescens* has emerged as a currently recognized pathogen for nosocomial infection in neonates, especially in epidemic form [4], which has also been reported in our experience. We collected 8 cases of *S. marcescens* bacteremia for 2 months. A similar report was published in 2010 by Gulcin Bayramoglu et al., comprising 9 cases, in neonatal intensive care unit over a period of 36 days [5].

Bacteriologically, *Serratia* is a Gram-negative bacillus belonging to the *Enterobacteriaceae* family, saprophytic genus, and widely found in the environment.

In *S. marcescens* outbreaks, sources of contamination reported in the literature were different: catheters, heparin solution, dialysis machine, propofol, liquid soap [6–11], and of course the manual transmission due to lack hospital hygiene.

The statistically significant risk factors of infection nosocomial to *Serratia* according to Al Jarousha and coworkers were low birth weight less than 1500 g, gestational age greater than 37 weeks, and mechanical ventilation [4]. Other risk factors that are common to all nosocomial infections are long hospital stay, antibiotic prescription, and the use of invasive methods: umbilical catheter, intubation, bladder catheterization, and parenteral nutrition [12]. However, our population was 75% premature and 87.5% artificially ventilated, the average weight recovered was 1850 g, and only one newborn had an umbilical vein catheter.

In our study, we isolated *Serratia* in blood cultures. This germ could be responsible for septicemia, pneumonia, conjunctivitis, urinary tract infections, and even gastroenteritis in newborns. In a study by Morillo et al., the clinical manifestations found in the ascending order were pneumonia, conjunctivitis, septicemia, and finally urinary tract infection [13]. Cerebromeningeal complications have recently been reported to be more frequent with *Serratia marcescens*, namely, meningitis, brain abscess, and empyema, and it is recommended to perform systematically brain imaging and more particularly transfontanellar ultrasound even in the absence of neurological signs [14].

With regard to the resistance of *S. marcescens* to antibiotics, high resistance to cephalosporins is often reported which can reach up to 100% [13, 15]. In our study, the cephalosporin resistance rate was lower and all strains were sensitive to imipenem and ciprofloxacin, which is consistent with the results reported by Al Jarousha who reported a sensitivity of 90% to imipenem and 76% to ciprofloxacin [4]. As for colistin, all our isolated strains were resistant concordant with the results of Buffet-Battailon et al. [16] and those of Adjidé et al. [15]. In addition to the resistance of *Serratia* to antibiotics, its severity is clear in light of the high mortality rates reported, which varies from 14.3% to 62.5% of cases [4, 5, 14, 15, 17–20] but in our study, we have a higher rate which was 62.5% (Table 3).

To cope with these epidemics, a number of measures were taken as soon as the first case is diagnosed, mainly the education of medical and paramedical staff on the importance of hand hygiene, the use of double protection by gloves, the technical and geographical isolation of the infected patients, and the disinfection of any surface or equipment likely to be contaminated. We achieved all these measures, which allowed a control of the epidemic at the end of August 2016.

## 5. Conclusion

Nosocomial infections with *Serratia marcescens* are beginning to take a large scale in neonatal intensive care unit, often in epidemic form. They are responsible for significant morbidity and mortality and pose the problem of acquiring antibiotic resistance. For this reason, prevention is the best method to fight them.

## Figures and Tables

**Table 1:** Patients' demographic data.

Patient no.	Diagnosis	Gestational age (weeks)	Weight (grams)	Method of delivery	Antibiotic prescription	Mechanical ventilation	Umbilical catheterization	Day of infection with *Serratia marcescens*	Outcome
1	Neonatal pulmonary infection	40.8	3600	Caesarean	Yes	Yes	None	12	Favorable
2	Hyaline membrane disease	33.2	1130	Vaginal delivery	Yes	Yes	None	7	Favorable
3	Hyaline membrane disease	33.9	1300	Vaginal delivery	Yes	Yes	None	3	Death
4	Neonatal pulmonary infection	34.6	1800	Vaginal delivery	Yes	Yes	None	6	Favorable
5	Hyaline membrane disease	35,3	1600	Vaginal delivery	Yes	Yes	None	5	Death
6	Prematurity	34.6	1400	Vaginal delivery	Yes	Yes	None	8	Death
7	Prematurity	35.9	1800	Vaginal delivery	Yes	None	None	5	Death
8	Neonatal pulmonary infection	40	2200	Vaginal delivery	Yes	Yes	None	11	Death

**Table 2:** Susceptibility to antibiotics of Serratia marcescens.

Antibiotic	Sensitivity of *S. marcescens*		
	Sensitivity	Intermediary	Resistance
Amoxicillin	None	None	100%
Piperacillin	75%	None	25%
Cefotaxime	87.5%	12.5%	None
Ceftazidime	87.5%	12.5%	None
Imipenem	100%	None	None
Ciprofloxacin	100%	None	None
Gentamicin	100%	None	None
Amikacin	100%	None	None
Colistin	None	None	100 %

**Table 3:** Summary of outbreaks caused by *S. marcescens* reported in the literature in comparison with our experience.

Series	Country/city	Year	Number of cases	Outcome
Al Jarousha et al. [4]	Palestine/Gaza	2008	159	70 died
Chemsi et al. [18]	Morocco/Casablanca	2013	9	5 died
Madide and Smith [14]	South Africa/Cape Town	2015	5	3 died
Polilli et al. [19]	Italy/Pescara	2011	6	2 died
Gulcin Bayramoglu et al. [5]	Turkey/Trabzon	2006	9	3
Walter Zingg et al. [20]	Switzerland/Geneva	2013	20	None died
Our cases	Morocco/Marrakesh	2016	8	5 died

## Data Availability

The data used to support the findings of this study are available from the corresponding author upon request.
